# Leisure Activity Variety and Brain Volume Among Community-Dwelling Older Adults: Analysis of the Neuron to Environmental Impact Across Generations Study Data

**DOI:** 10.3389/fnagi.2021.758562

**Published:** 2021-11-30

**Authors:** Ai Iizuka, Hiroshi Murayama, Masaki Machida, Shiho Amagasa, Shigeru Inoue, Takeo Fujiwara, Yugo Shobugawa

**Affiliations:** ^1^Research Team for Social Participation and Community Health, Tokyo Metropolitan Institute of Gerontology, Tokyo, Japan; ^2^Department of Preventive Medicine and Public Health, Tokyo Medical University, Tokyo, Japan; ^3^Department of Global Health Promotion, Tokyo Medical and Dental University, Tokyo, Japan; ^4^Department of Active Ageing, Niigata University Graduate School of Medical and Dental Sciences, Niigata, Japan

**Keywords:** brain reserve, brain volume, hippocampus, gray matter, leisure activity, magnetic resonance imaging, older people

## Abstract

**Background:** Recent findings indicate that leisure activity (LA) delays cognitive decline and reduces the risk of dementia. However, the association between LA and brain volume remains unclear. This study aimed to examine the association between LA variety and brain volume with a focus on the hippocampus and gray matter.

**Methods:** Data were obtained from the baseline survey of the Neuron to Environmental Impact across Generations study, which had targeted community-dwelling older adults living in Niigata, Japan. We divided LAs into 10 categories, and counted the number of categories of activities in which the participants engaged. We classified them as follows: 0 (i.e., no activity), 1, 2, or ≥ 3 types. Brain volume was assessed through magnetic resonance imaging, and hippocampal and gray matter volumes were ascertained.

**Results:** The sample size was 482. Multiple linear regression analysis showed that hippocampal and gray matter volumes were significantly greater among participants with ≥ 3 types of LAs than among their no-activity counterparts. Hippocampal volume was significantly greater among those who engaged in one type of LA than among those who engaged in no such activity. Sex-stratified analysis revealed that hippocampal volumes were significantly greater among males who engaged in ≥ 3 types of LAs and one type of LA. However, no such association was found among females.

**Conclusion:** The present findings suggest that engaging in a wide range of LAs is related to hippocampal and gray matter volumes. Furthermore, there was a sex difference in the association between LA variety and brain volume.

## Introduction

Non-pharmacological approaches are expected to delay cognitive decline and reduce the risk of dementia. Leisure activity (LA), which refers to activities that “individuals engage in for enjoyment or well-being that are independent of activities of daily living” (Leung et al., 2011) is one of the methods included in non-pharmacological approaches. Previous studies have reported that engaging in LA in later life maintains cognitive function and reduces the risk of dementia (Verghese et al., 2003, 2006; Fallahpour et al., 2016; Yates et al., 2016). The underlying mechanism is considered to be related to the accumulation of cognitive reserves (Stern, 2002, 2009, 2012).

There is another concept called brain reserve, which explains why individuals differ in their susceptibility to cognitive aging and the conditions associated with Alzheimer’s disease (Fratiglioni and Wang, 2007; Valenzuela, 2008; Stern, 2012). Brain volume, which is a typical index of brain reserve, is regarded as a correlate of cognitive function and the risk of dementia (Katzman et al., 1988; Fratiglioni and Wang, 2007; Valenzuela, 2008; Stern, 2012). Several studies have shown that larger hippocampal and gray matter volumes are associated with higher cognitive function and a lower risk of dementia (MacLullich et al., 2002; Fotenos et al., 2005; Brickman et al., 2007; Taki et al., 2011; Teipel et al., 2013; Vaughan et al., 2014; Hilal et al., 2015). However, the relationship between LA and brain volume has not been examined among older adults.

Engaging in a broad (rather than a narrow) range of LAs may strongly affect brain volume as it may lead to strong stimulation of the brain and enhance neuroplasticity. Several studies have found that engaging in a broader range of LAs lowers the risk of dementia (Leung et al., 2011; Kozono et al., 2016; Ling et al., 2020), and broader LA contents are associated with higher cognitive test scores (Ihle et al., 2015). Moreover, in Japan, it has been found that engaging in a broader range of LAs promotes stability, improves mental health (e.g., higher life satisfaction called ikigai) (Haraoka, 2004), increases resilience (Matsunaka et al., 2019), and reduces the risk of frailty (Fushiki et al., 2012). Thus, engaging in a broader range of LAs has a positive effect on health; therefore, we hypothesized that this may also hold true for brain volume.

Accordingly, we examined the association between LA variety and brain volume with a focus on the hippocampus and gray matter among community-dwelling older adults in Japan.

## Materials and Methods

### Study Sample and Data Collection

Data were obtained from the baseline survey of the Neuron to Environmental Impact across Generations (NEIGE) study conducted in Tokamachi City, Niigata, Japan, in 2017. Tokamachi City is a rural area located approximately 180 km northwest of Tokyo. The population of the city was 51,964, and the proportion of older adults was 36.9% as of January 31, 2020. Of all those aged 65–84 years who were living in Tokamachi City, 1346 individuals who were not recipients of long-term care insurance and were not admitted to a hospital or nursing home were randomly selected from a resident registry. A total of 527 individuals, who agreed to participate in the NEIGE study, were enrolled in the survey. A detailed profile of this study (study concept, design, and sample) is elsewhere (Shobugawa et al., 2020).

### Ethical Approval

The study protocol was approved by The University Ethics Committee (Niigata University and Tokyo Medical University; approval number: 2666 and 3921). We explained the purpose, methods, and ethical considerations of this study and obtained written informed consent from all participants based on the Declaration of Helsinki before enrolment.

### Measures

#### Leisure Activity Variety

We developed original questions about LA. First, the participants responded with either “yes” or “no” to indicate whether they were currently engaged in LA. If the participant’s response was “yes,” they were further asked to indicate whether they engaged in any of the following 20 activities: grand golf, golf, pachinko (Typical Japanese gambling device resembling slot machine), gymnastics or tai chi, walking or jogging, computer use, reading books, playing board games, painting, fishing, karaoke, dancing, craftwork, calligraphy, tea ceremony or flower arrangement, crop work, gardening, photography, traveling, and others. These activities are regarded as popular LAs in Japan based on previous studies and literatures (Kozono et al., 2016; Iwasa and Yoshida, 2018; Japan Productivity Centre, 2019). Participants who chose the “Others” option were asked to specifically describe the activities in which they engaged. The information about LA was collected with a self-reported questionnaire.

After data were collected, we classified the items (including “Others” responses) into the following 10 categories of LAs: (1) physical activities (walking, jogging, gymnastics, tai chi, dancing, fishing, golf, ground golf, climbing, skiing, swimming, tennis, table tennis, volleyball, baseball, and cycling); (2) gaming (board games, crossword puzzles, and billiards); (3) traveling (traveling and driving); (4) creative activities (painting, craftwork, collecting stamps, and carpentry); (5) cultural activities (flower arrangement, tea ceremony, calligraphy, and poetry, including Japanese haiku, tanka, and shigin); (6) developmental activities (reading books, reading aloud, studying history, and attending public lectures); (7) agricultural activities (crop work and gardening); (8) singing (chorus and karaoke); (9) gambling (pachinko and stock investment); and (10) technology use (computer use and photography). We developed these categories based on a review of previous studies conducted in Japan (Kozono et al., 2016; Iwasa and Yoshida, 2018). Finally, we counted the number of categories of LAs reported by each participant, and divided them into quartiles [0 type (i.e., = no activity), 1 type, 2 types, or ≥ 3 types out of 10].

#### Brain Volume

Each participant underwent magnetic resonance imaging (MRI) at the Niigata Prefectural Tokamachi Hospital. We used a 1.5 Tesla scanner (MAGNETOM Avanto fit, Siemens, Germany) in three-dimensional mode with the following parameters: repetition time = 1,700 ms; echo time = 4.31 ms; inversion time = 800 ms; flip angle = 15°; slice thickness = 1.25 mm; field of view = 230 × 230; matrix size = 256 × 256; and number of slices = 144. Segmentation and volume calculations were undertaken using FreeSurfer Version 6.0^1^.

For this procedure, we removed non-brain tissue (e.g., brain skull), normalized the voxel intensities, and labeled the volumes of each segmentation using FreeSurfer. Hippocampal volume, total gray matter volume, and intracranial volume were automatically derived and labeled. Hippocampal volume was separately determined for the left and right hemispheres, and the total volume of the left and right hippocampus (total hippocampal volume) was used in the analysis.

#### Covariates

Sociodemographic factors, health conditions, physical activity, and social interaction served as covariates. The information regarding the covariates was collected with a self-reported questionnaire and interviews conducted by trained staff. The assessed sociodemographic factors included age, sex, years of education (≤ 9 years or ≥ 10 years), annual household income (< 2.5 million yen, 2.5–3.9 million yen, 4.0–6.9 million yen, or ≥ 7.0 million yen), current occupational status (employed or unemployed).

The assessed health conditions included comorbidities and depressive mood. Information on comorbidities was obtained through interviews conducted by medical doctors or nurses. Hypertension, diabetes, cardiovascular disease, cerebrovascular disease, and neuropsychiatric disorders served as covariates. Depressive mood was assessed using the Japanese version of the Geriatric Depression Scale (GDS): Short Form (Sheikh and Yesavage, 1986; Sugishita and Asada, 2009). The GDS consisted of 15 items, and respondents answered dichotomous questions. The responses were summed; total scores can range from 0 to 15. The Cronbach’s alpha of this scale was 0.77 in this study. We used a cut-off point of ≥ 6, which indicated a depressive mood (Sugishita and Asada, 2009).

Physical activity was assessed using an accelerometer, namely, the Active style Pro HJA-750C (Omron Healthcare, Kyoto, Japan). The participants were asked to wear the accelerometer over their waist on an elasticated belt for seven consecutive days while awake, and average daily amount of moderate-to-vigorous (i.e., ≥ 3.0 metabolic equivalents) physical activity served as covariates. Participants who did not have valid data for more than 4 days were excluded. A more detailed description of the accelerometer is available elsewhere (Oshima et al., 2010; Ohkawara et al., 2011; Park et al., 2017).

Social interaction was assessed based on the frequency of meeting friends and acquaintances. The frequency of meeting friends and acquaintances was enquired with a question comprising six categories (≥ 4 days/week, 2–3 days/week, 1 day/week, 1–3 days/month, several times/year, and none). We divided the participants based on this frequency into two groups: < 1 day/week and ≥ 1 day/week.

In addition to these covariates, we assessed the participants’ cognitive status using the Mini-Mental State Examination-Japanese version (MMSE-J) (Sugishita et al., 2016). The MMSE-J consists of 11 questions, and total scores can range from 0 to 30. We used the MMSE-J scores to examine participant characteristics, but this variable was not included in the regression model.

### Statistical Analysis

Participant’s characteristics by categories of LAs were compared using one-way analysis of variance for continuous variables and chi-square test for categorical variables.

We conducted multiple linear regression analysis to examine the association between LA variety and brain volume. We separately entered hippocampal and gray matter volumes as dependent variables; the different categories of LAs (divided into quartiles) served as the independent variable. The LA categories were entered into the regression models as dummy variables, and the no-activity group (i.e., 0 types) served as the reference. Model 1 was adjusted for age, sex, and years of education, which are generally included as covariates in studies on LA, and intracranial volume. Model 2 was additionally adjusted for annual household income, occupational status, comorbidities, GDS score, physical activity, and social interaction which are potential confounding factors. Further, to examine sex differences, the data were stratified by sex, and the analysis was separately conducted for males and females.

For reference, we conducted the following two analyses: (1) stratification by left and right hippocampus as some studies indicated differential effect of health behaviors on brain volume by the brain side (Firth et al., 2018; Machida et al., 2021; Vujic et al., 2021) and (2) adding an interaction term between LA variety and comorbidities/mental health on brain volume to consider the possibility that the relationship between LA variety and brain volume varies by disease and mental health condition because chronic disease and mental health can be possible confounders (Campbell et al., 2004; Firbank et al., 2007; Callisaya et al., 2019).

The associations were examined by computing regression coefficients (b) and 95% confidence intervals (CIs). All analyses were conducted using IBM Statistical Package for the Social Sciences version 23 (IBM Inc., Chicago, IL, United States).

## Results

Of the 527 participants, we excluded 27 individuals whose MRI data were not of sufficient quality and 18 others with missing data for any of the covariates. Thus, the data of 482 participants were analyzed.

Participant characteristics are presented in Table 1. Their average age was 73.2 years (standard deviation = 5.4), and 47.3% of them were males. With regard to years of education, 37.1% had received less than 9 years of education. Approximately half of the participants had hypertension (52.9%), and 18.0% of them obtained scores ≥ 6 on the GDS. The average MMSE-J score was 27.1 (standard deviation = 2.5), and three participants reported that they had been diagnosed with dementia. The percentages of individuals with 0, 1, 2, and ≥ 3 types of LAs were 15.1, 25.1, 26.3, and 33.4%, respectively.

**TABLE 1:** Participant characteristics (*N* = 482).

Variable	Category	Mean (SD)	*n*	%
Age (years)		73.2 (5.4)		
Sex	Males		228	47.3
	Females		254	52.7
Years of education	≤9		179	37.1
	≥10		303	62.9
Annual household income (million yen)	<2.5		135	28.0
	2.5–3.9		127	26.3
	4.0–6.9		122	25.3
	≥7.0		98	20.3
Current occupational status	Employed		201	41.7
	Unemployed		281	58.3
Comorbidities				
Hypertension	Having		255	52.9
	Not having		227	47.1
Diabetes	Having		87	18.0
	Not having		395	82.0
Cardiovascular disease	Having		93	19.3
	Not having		389	80.7
Cerebrovascular disease	Having		35	7.3
	Not having		447	92.7
Neuropsychiatric disorder	Having		21	4.4
	Not having		461	95.6
GDS score	≤5		404	83.8
	≥ 6		78	18.0
Physical activity^a^ (METs-hours per day)		3.0 (2.3)		
MMSE-J score		27.1 (2.5)		
Social interaction	<1 day/week		202	41.9
	≥1 day/week		280	58.1
Leisure activity variety (type)^b^	0		73	15.1
	1		121	25.1
	2		127	26.3
	≥3		161	33.4
Brain volume (mm^3^)				
Intracranial volume		1433189.2 (152127.6)		
Total hippocampal volume		7213.2 (775.7)		
Gray matter volume		569911.0 (46847.1)		

*GDS, Japanese version of the Geriatric Depression Scale: Short Form; METs, Metabolic equivalents; MMSE-J, Mini-Mental State Examination-Japanese version; SD, standard deviation.*

*^a^Level of physical activity ≥ 3.0 METs.*

*^b^Number of categories of leisure activities.*

Table 2 shows a comparison between the participant’s characteristics. There were significant differences among years of education, number of participants who had neuropsychiatric disorder, GDS score, MMSE-J score, and social interaction between categories of LAs (all *p* < 0.05).

**TABLE 2:** The comparison of the participant’s characteristics by categories of leisure activity (*N* = 482).

Variable		Leisure activity variety				*p* ^a^
	
		0 type	1 type	2 types	≥ 3 types	
Age (years)	Mean (SD)	72.6 (5.4)	73.9 (6.0)	73.7 (5.5)	72.6 (5.0)	0.126
Sex (males)	%	43.8	39.6	50.3	52.1	0.158
Years of education (≤ 9 years)	%	46.5	43.8	33.0	31.0	0.036
Annual household income (< 2.5 million yen)	%	26.0	30.5	26.7	27.9	0.912
Current occupational status (Employed)	%	35.6	42.1	39.3	45.9	0.456
Comorbidities (Having)						
Hypertension	%	53.4	53.7	45.6	57.7	0.237
Diabetes	%	17.8	23.1	13.3	18.0	0.262
Cardiovascular disease	%	15.0	19.0	22.0	19.2	0.691
Cerebrovascular disease	%	5.4	6.6	4.7	10.5	0.237
Neuropsychiatric disorder	%	6.8	3.3	7.8	1.2	0.030
GDS score (≥ 6)	%	28.7	18.1	18.8	6.8	<0.001
Physical activity (METs-hours per day)	Mean (SD)	3.2 (2.2)	3.1 (2.8)	2.6 (2.0)	3.2 (2.1)	0.134
Social interaction (≥ 1 day/week)	%	53.4	52.8	53.5	67.7	0.027
MMSE-J score	Mean (SD)	26.3 (3.0)	27.0 (2.3)	27.0 (2.6)	27.4 (2.2)	0.012
Brain volume						
Total hippocampal volume (mm^3^)	Mean (SD)	7054.1 (806.9)	7187.1 (832.7)	7203.6 (726.2)	7312.5 (747.4)	0.118
Gray matter volume (mm^3^)	Mean (SD)	563118.5 (47214.4)	562766.8 (46939.9)	574726.9 (41974.8)	574561.3 (49544.9)	0.063

*GDS, Japanese version of the Geriatric Depression Scale short-form; MMSE-J, Mini-Mental State Examination, Japanese version; SD, Standard deviation.*

*^a^p-values indicate the results of one-way analysis of variance for continuous variables and chi-square test for categorical variables.*

Table 3 presents the association between LA variety and brain volume. Participants with ≥ 3 types of LAs had significantly greater total hippocampal and gray matter volumes than their no-activity counterparts, when age, sex, and years of education were controlled for in Model 1 (b [95% CI] = 217.3 [46.3–388.4] for total hippocampal volume and 7954.6 [1197.0–14712.1] for total gray matter volume). This association remained significant even after the model was additionally adjusted for sociodemographic factors, health conditions, physical activity, and social interaction (Model 2; 216.8 [39.1–394.5] for total hippocampal volume, and 7466.4 [490.3–14442.5] for total gray matter volume).

**TABLE 3:** Association between leisure activity variety and brain volume: Results of multiple regression analysis.

Model	Leisure activity variety	Total hippocampal volume		Gray matter volume	
		b	95% CI	b	95% CI
Model 1	0 type	Ref.		Ref.	
	1 type	213.5	(34.3, 392.8)	3431.5	(−3649.7, 10512.8)
	2 types	86.2	(−92.8, 265.2)	1176.9	(−5897.9, 8251.8)
	≥ 3 types	217.3	(46.3, 388.4)	7954.6	(1197.0, 14712.1)
		*p* for trend = 0.085		*p* for trend = 0.027	
Model 2	0 type	Ref.		Ref.	
	1 type	205.9	(23.8, 388.1)	3321.0	(−3830.5, 10472.7)
	2 types	83.2	(−98.5, 264.9)	259.7	(−6876.4, 7396.0)
	≥ 3 types	216.8	(39.1, 394.5)	7466.4	(490.3, 14442.5)
		*p* for trend = 0.100		*p* for trend = 0.057	

*CI, confidential interval; Ref., reference.*

*Model 1: adjusted for age, sex, years of education, and intracranial volume.*

*Model 2: additionally adjusted for current occupational status, annual household income, comorbidities (hypertension, diabetes, cardiovascular disease, cerebrovascular disease, and neuropsychiatric disorder), the Japanese version of the Geriatric Depression Scale: Short Form, physical activity, and social interaction.*

Further, participants who engaged in one type of LA had greater total hippocampal volumes than their no-activity counterparts in both Models 1 (213.5 [34.3–392.8]) and 2 (205.9 [23.8–388.1]).

We separately conducted multiple regression analysis using data collected from males and females (Table 4). There were sex differences in the emergent associations. Males who engaged in ≥ 3 types of LAs and one type of LA had greater total hippocampal volume in Models 1 (≥ 3 types: 329.3 [61.3–597.4]; one type: 352.4 [57.1–647.7]) and 2 (≥ 3 types: 319.0 [38.3–599.8]; one type: 320.6 [12.8–628.4]). However, there were no significant associations among females. In addition, stratified analysis revealed that there was no significant association between LA variety and total gray matter volume among either males or females.

**TABLE 4:** Association between leisure activity variety and brain volume by sex.

Model	Leisure activity variety	Total hippocampal volume		Gray matter volume	
		b	95% CI	b	95% CI
**Males**					
Model 1	0 type	Ref.		Ref.	
	1 type	352.4	(57.1, 647.7)	4840.1	(−6749.6, 16426.9)
	2 types	279.9	(−3.2, 563.1)	3360.1	(−7750.5, 14470.7)
	≥ 3 types	329.3	(61.3, 597.4)	9575.7	(−942.0, 20093.6)
		*p* for trend = 0.083		*p* for trend = 0.077	
Model 2	0 type	Ref.		Ref.	
	1 type	320.6	(12.8, 628.4)	5790.4	(−6236.4, 17817.3)
	2 types	266.8	(−28.0, 561.8)	1728.9	(−9795.8, 13253.7)
	≥ 3 types	319.0	(38.3, 599.8)	8438.9	(−2530.3, 19408.1)
		*p* for trend = 0.090		*p* for trend = 0.191	
**Females**					
Model 1	0 type	Ref.		Ref.	
	1 type	117.8	(-105.9, 341.5)	2338.1	(−6667.8, 11344.0)
	2 types	−63.0	(-292.6, 166.6)	−395.7	(−9640.1, 8848.5)
	≥ 3 types	139.6	(-82.1, 361.4)	6533.1	(−2395.8, 15462.1)
		*p* for trend = 0.495		*p* for trend = 0.186	
Model 2	0 type	Ref.		Ref.	
	1 type	101.1	(−126.4, 328.6)	1619.7	(−7482.0, 10721.5)
	2 types	−55.5	(−288.5, 177.4)	−963.9	(−10285.8, 8357.9)
	≥ 3 types	109.8	(−122.7, 342.5)	5656.3	(−3652.1, 14964.8)
		*p* for trend = 0.692		*p* for trend = 0.287	

*CI, confidential interval; Ref., reference.*

*Model 1: adjusted for age, sex, years of education, and intracranial volume.*

*Model 2: additionally adjusted for annual household income, current occupational status, comorbidities (hypertension, diabetes, cardiovascular disease, cerebrovascular disease, and neuropsychiatric disorder), the Japanese version of the Geriatric Depression Scale: Short Form, physical activity, and social interaction.*

For reference, the results of a separate analysis conducted for the left and right hippocampal volumes are present in Supplementary Material A. Participants with ≥ 3 types of LAs had a significantly greater right and left hippocampal volumes than their no-activity counterparts. The participants engaged in one LA type had an enlarged hippocampal volume only on the left side. In addition, the interaction of diabetes × LA variety was robust (Supplementary Material B), and the association between LA variety and brain volume was stronger in participants with diabetes.

These results did not change significantly when we excluded the three participants who had been diagnosed with dementia (mean age = 69 years; two males and one female) from the analysis due to concerns about recall bias (Supplementary Materials C, D).

## Discussion

This study examined the association between LA variety and brain volume, with a focus on the hippocampus and gray matter, among community-dwelling Japanese older adults. The results showed that hippocampal and gray matter volumes were significantly greater among those who engaged in ≥ 3 types of LAs than those who engaged in no such activity. Moreover, total hippocampal volumes were greater among those who engaged in one type of LA than among those who engaged in no such activity. Furthermore, there was a sex difference in the association between LA variety and brain volume, which was more pronounced among males than among females.

Total hippocampal and gray matter volumes are associated with the risk of dementia (Du et al., 2001; Grundman et al., 2002; Cardenas et al., 2003; Taki et al., 2011; Teipel et al., 2013; Hilal et al., 2015). In this study, those who engaged in ≥ 3 types of LAs had significantly greater hippocampal and gray matter volumes than those who engaged in no such activity. Previous studies have reported that total hippocampal volume atrophies by approximately 0.98–1.12% annually and that total gray matter volume atrophies by approximately 0.30% because of natural aging (Taki et al., 2011; Fraser et al., 2015). In this study, those who engaged in ≥ 3 types of LAs had approximately 3.0% greater total hippocampal volume (217 mm^3^/7213 mm^3^) and approximately 1.3% greater total gray matter volume (7954 mm^3^/569911 mm^3^) than their no-activity counterparts. This indicates that there is a difference in the brain volume equivalent to 3–4 years of atrophy in those who engage in ≥ 3 types of LAs than those who engage in no such activity. According to the evidence for the basic biological consequences of environmental enrichment, more complex and stimulating environments induce neural and synaptic structural changes, such as dendritic arborization and synaptogenesis (West and Greenough, 1972; Kempermann et al., 1997; Nithianantharajah and Hannan, 2006), and this principle can be extended to human life itself (Queen et al., 2020). As a mechanism to promote synaptogenesis, an enriched environment increases brain-derived neurotrophic factor (BDNF) and binds it to receptors (TrkB) in the hippocampus and cerebral cortex, resulting in long-term potentiation and promoting neurogenesis (Kempermann et al., 2002; Rossi et al., 2006). It is regarded as a phenomenon that applies to humans as well, since previous studies have shown that exercise, certain types of games, and social activities increase BDNF and maintain the hippocampal volume and gray matter volume (Erickson et al., 2011; Carlson et al., 2015; Lin et al., 2015). Engagement in a wide range of LAs involves exposure to more stimulating and complex environments; in this regard, the present findings can be considered logically reasonable.

There was a similar association between LA variety and hippocampal volumes in males; however, it was not significant among females. In Japan, older females tend to have strong social relationships and actively engage not only in LAs but also other activities such as socializing with neighbors and friends and housework than older males (Hatanaka and Tanaka, 1999; Hirai et al., 2005). Therefore, LA may have had a relatively small effect and may not have been associated with brain volume among females.

There were no significant differences in the brain volumes of participants who engaged in 2 types of LAs and their no-activity counterparts. However, participants who engaged in one type of LA had greater total hippocampal volumes than those who engaged in no such activity. This was an unexpected finding as it was unlikely to be a matter of statistical power, such as sample size. In an intervention study involving cognitive activities, it was found that focusing on one type of intervention (photography) rather than a combination of 2 types of interventions (photography and craftwork) was more effective in improving episodic memory (Park et al., 2014). Those who participated in only one type of intervention may have focused on their activities more keenly, and this may have had a greater effect on episodic memory than participation in 2 types of intervention. Similarly, even in this study, those who engaged in one type of LA may have done so more intensively than those who engaged in 2 types of LAs; this may have affected their hippocampal volume. Conversely, people who engaged in ≥ 3 types of LAs may show a greater effect on the total hippocampal and gray matter volumes as they have engaged in LAs more intensively or frequently and have received more stimulation than those who engaged in 1 or 2 types of LAs. However, it is not possible to reach a complete conclusion. It is also possible that there is a sample bias, such as the presence of some special characteristics in those who engaged in 2 types of LAs. Therefore, further examination is required to reveal the mechanisms in the future.

This study yielded novel findings about the association between LA variety and brain volume; however, this study also has some limitations. First, we did not assess LAs in great detail. Indeed, the effect of LA on brain volume may differ depending on LA frequency, intensity, location, and group members. Such information should be collected in future studies. In addition, although we focused on LA variety and classified LAs into 10 categories on the basis of the types of LA, there is diversity even within the same type of LA when examined in detail. Therefore, not only the LA variety but also the number of LAs is important. We intend to investigate the relationship between the number of each LA and brain volume in a future study. Second, this study was conducted in a rural area; therefore, care should be taken while generalizing the findings. Third, the fact that the association between LA and hippocampal volume was different in the left and right sides, and the association was stronger in people with diabetes needs to be investigated in future studies. Finally, because this study adopted a cross-sectional design, we could not determine if LA variety results in greater brain volume or if people with greater brain volumes engage in a broader range of LAs. Therefore, future studies should examine the association between LA and brain volume longitudinally.

## Conclusion

The present findings suggest that engaging in a wide range of LAs is related to greater total hippocampal and gray matter volumes among community-dwelling older adults. Moreover, there was a sex difference in the association between LA variety and brain volume. Further research is needed to longitudinally examine the causal relationship between LA variety and brain volume.

## Data Availability Statement

The raw data supporting the conclusions of this article will be made available by the authors, without undue reservation.

## Ethics Statement

The studies involving human participants were reviewed and approved by the Niigata University and Tokyo Medical University. The patients/participants provided their written informed consent to participate in this study.

## Author Contributions

AI and HM: conceptualization, formal analysis, and methodology. MM, SA, SI, TF, and YS: data curation and project administration. HM, MM, SA, SI, TF, and YS: investigation. YS: supervision. AI and YS: writing–original draft. All authors have read and approved the published version of the manuscript.

## Conflict of Interest

This study received funding from the Pfizer Health Research Foundation. The funder was not involved in the study design, collection, analysis, interpretation of data, the writing of this article or the decision to submit it for publication. The authors declare that the research was conducted in the absence of any commercial or financial relationships that could be construed as a potential conflict of interest.

## Publisher’s Note

All claims expressed in this article are solely those of the authors and do not necessarily represent those of their affiliated organizations, or those of the publisher, the editors and the reviewers. Any product that may be evaluated in this article, or claim that may be made by its manufacturer, is not guaranteed or endorsed by the publisher.

## Footnotes

1
http://surfer.nmr.mgh.harvard.edu
