# Supplementary material for: Leisure Activity Variety and Brain Volume Among Community-Dwelling Older Adults: Analysis of the Neuron to Environmental Impact Across Generations Study Data
Source: Front Aging Neurosci. 2021 Nov 30;13:758562. doi: 10.3389/fnagi.2021.758562 (PMC8669795; doi:10.3389/fnagi.2021.758562)
Supplement: Supplementary file 1 [file Data_Sheet_1.docx]

Supplementary Material

Supplementary A. Association between leisure activity variety and the left and right hippocampus.

| Model | Leisure activity variety | Left hippocampal volume | |  | Right hippocampal volume | |
| --- | --- | --- | --- | --- | --- | --- |
|  |  | B | 95%CI |  | b | 95%CI |
| Model 1 | 0 type | Ref. | |  | Ref. | |
|  | 1 type | 119.3 | (26.8, 211.8) |  | 94.2 | (-5.7, 194.2) |
|  | 2 types | 54.4 | (-37.9, 146.9) |  | 31.7 | (-68.2, 131.6) |
|  | ≥3 types | 107.8 | (19.5, 196.1) |  | 109.5 | (14.1, 204.9) |
|  |  | *p* for trend = 0.125 | |  | *p* for trend = 0.094 | |
| Model 2 | 0 type | Ref. | |  | Ref. | |
|  | 1 type | 113.6 | (19.8, 207.3) |  | 92.3 | (-9.1, 193.9) |
|  | 2 types | 47.3 | (-46.2, 140.8) |  | 35.8 | (-65.4, 137.2) |
|  | ≥3 types | 108.4 | (16.9, 199.8) |  | 108.4 | (9.3, 207.5) |
|  |  | *p* for trend = 0.135 | |  | *p* for trend = 0.115 | |

CI = confidential interval; Ref. = reference.

Model 1: adjusted for age, sex, years of education, and intracranial volume.

Model 2: additionally adjusted for current occupational status, annual household income, comorbidities (hypertension, diabetes, cardiovascular disease, cerebrovascular disease, and neuropsychiatric disorder), the Japanese version of the Geriatric Depression Scale: Short Form, physical activity, and social interaction.

Supplementary B. Analysis of the interaction between leisure activity variety and diabetes on brain volume.^a^

|  | | Total hippocampal volume | |  | Gray matter volume | |
| --- | --- | --- | --- | --- | --- | --- |
|  |  | b | 95% CI |  | B | 95% CI |
| LA variety | 0 type | Ref. | |  | Ref. | |
|  | 1 type | 206.9 | (25.4, 388.4) |  | 3161.7 | (-4028.9, 10352.4) |
|  | 2 types | 85.0 | (-96.0, 266.1) |  | 116.6 | (-7057.0, 7290.3) |
|  | ≥3 types | 220.5 | (43.9, 397.1) |  | 7422.7 | (426.9, 14418.4) |
|  |  |  | |  |  | |
| Interaction  (LA variety ×Diabetes) | 0 type × Diabetes | Ref. | |  | Ref. | |
|  | 1 type × Diabetes | 564.4 | (108.5, 1020.3) |  | -459.4 | (-18520.5, 17601.6) |
|  | 2 types × Diabetes | 512.1 | (24.3, 999.9) |  | -5775.7 | (-25098.1, 13546.7) |
|  | 3 types × Diabetes | 664.1 | (215.9, 1112.3) |  | -3931.5 | (-21684.9, 13821.8) |

CI = confidential interval; Ref. = reference; LA = leisure activity.

^a^ Adjusted for age, sex, years of education, intracranial volume, current occupational status, annual household income, comorbidities (hypertension, diabetes, cardiovascular disease, cerebrovascular disease, and neuropsychiatric disorder), the Japanese version of the Geriatric Depression Scale: Short Form, and physical activity, and social interaction.

Supplementary C. Association between leisure activity variety and brain volume: results of the analysis that excluded participants with dementia.

| Model | Leisure activity variety | Total hippocampal volume | |  | Gray matter volume | |
| --- | --- | --- | --- | --- | --- | --- |
|  |  | b | 95% CI |  | b | 95% CI |
| Model 1 | 0 type | Ref. | |  | Ref. | |
|  | 1 type | 216.6 | (36.5, 396.8) |  | 4065.9 | (-3045.9, 11177.7) |
|  | 2 types | 83.9 | (-95.6, 263.5) |  | 1727.4 | (-5363.3, 8818.1) |
|  | ≥3 types | 224.4 | (52.7, 396.1) |  | 8590.6 | (1812.1, 15369.2) |
|  |  | *p* for trend = 0.074 | |  | *p* for trend = 0.019 | |
| Model 2 | 0 type | Ref. | |  | Ref. | |
|  | 1 type | 207.0 | (24.4, 389.7) |  | 3826.9 | (-3357.4, 11011.2) |
|  | 2 types | 81.6 | (-100.4, 263.6) |  | 836.3 | (-6324.3, 7996.9) |
|  | ≥3 types | 223.8 | (45.9, 401.8) |  | 8024.2 | (1025.3, 15023.1) |
|  |  | *p* for trend = 0.085 | |  | *p* for trend = 0.042 | |

CI = confidential interval; Ref. = reference.

Model 1: adjusted for age, sex, years of education, and intracranial volume.

Model 2: additionally adjusted for current occupational status, annual household income, comorbidities (hypertension, diabetes, cardiovascular disease, cerebrovascular disease, and neuropsychiatric disorder), the Japanese version of the Geriatric Depression Scale: Short Form, physical activity, and social interaction.

Supplementary D. Association between leisure activity variety and brain volume by sex: results of the analysis that excluded participants with dementia.

| Model | Leisure activity variety | Total hippocampal volume | |  | Gray matter volume | |
| --- | --- | --- | --- | --- | --- | --- |
|  |  | b | 95% CI |  | b | 95% CI |
| Males |  |  |  |  |  |  |
| Model 1 | 0 type | Ref. | |  | Ref. | |
|  | 1 type | 347.1 | (50.5, 643.8) |  | 4903.7 | (-6789.7, 16597.2) |
|  | 2 types | 266.8 | (-16.7, 550.4) |  | 3223.3 | (-7954.9, 14401.6) |
|  | ≥3 types | 330.6 | (62.6, 598.5) |  | 9592.1 | (-970.1, 20154.5) |
|  |  | *p* for trend = 0.077 | |  | *p* for trend = 0.079 | |
| Model 2 | 0 type | Ref. | |  | Ref. | |
|  | 1 type | 305.9 | (-2.7, 614.7) |  | 5389.6 | (-6736.0, 17515.3) |
|  | 2 types | 237.8 | (-58.3, 534.1) |  | 1151.5 | (-10482.4, 12785.5) |
|  | ≥3 types | 307.9 | (27.3, 588.4) |  | 8186.5 | (-2832.3, 19204.5) |
|  |  | *p* for trend = 0.101 | |  | *p* for trend = 0.200 | |
| Females |  |  |  |  |  |  |
| Model 1 | 0 type | Ref. | |  | Ref. | |
|  | 1 type | 127.2 | (-97.9, 352.4) |  | 3367.5 | (-5635.4, 12370.4) |
|  | 2 types | -56.7 | (-287.3, 173.8) |  | 709.7 | (-8510.4, 9930.0) |
|  | ≥3 types | 149.4 | (-74.1, 372.9) |  | 7654.2 | (-1284.4, 16592.9) |
|  |  | *p* for trend = 0.464 | |  | *p* for trend = 0.127 | |
| Model 2 | 0 type | Ref. | |  | Ref. | |
|  | 1 type | 109.6 | (-119.0, 338.3) |  | 2358.4 | (-6758.2, 11475.1) |
|  | 2 types | -43.1 | (-277.8, 191.5) |  | 39.4 | (-9318.6, 9397.5) |
|  | ≥3 types | 119.8 | (-114.5, 354.1) |  | 6529.8 | (-2813.4, 15873.1) |
|  |  | *p* for trend = 0.634 | |  | *p* for trend = 0.215 | |

CI = confidential interval; Ref. = reference.

Model 1: adjusted for age, sex, years of education, and intracranial volume.

Model 2: additionally adjusted for annual household income, current occupational status, comorbidities (hypertension, diabetes, cardiovascular disease, cerebrovascular disease, and neuropsychiatric disorder), the Japanese version of the Geriatric Depression Scale: Short Form, physical activity, and social interaction.
